# Correlation Between TNFAIP2 Gene Polymorphism and Prediction/Prognosis for Gastric Cancer and Its Effect on TNFAIP2 Protein Expression

**DOI:** 10.3389/fonc.2020.01127

**Published:** 2020-07-24

**Authors:** Fang Guo, Qian Xu, Zhi Lv, Han-Xi Ding, Li-Ping Sun, Zhen-Dong Zheng, Yuan Yuan

**Affiliations:** ^1^Tumor Etiology and Screening Department of Cancer Institute and General Surgery, The First Hospital of China Medical University, Shenyang, China; ^2^Department of Oncology, PLA Cancer Center, General Hospital of Northern Theater Command, Shenyang, China; ^3^Key Laboratory of Cancer Etiology and Prevention in Liaoning Education Department, The First Hospital of China Medical University, Shenyang, China; ^4^Key Laboratory of GI Cancer Etiology and Prevention in Liaoning Province, The First Hospital of China Medical University, Shenyang, China

**Keywords:** gastric cancer, TNFAIP2, SNP, prediction, prognosis

## Abstract

**Objective:** TNFAIP2 is a novel gene induced by TNF-α and participates in inflammatory reaction and tumor angiogenesis. This study aims to understand the correlation between TNFAIP2 gene polymorphism and prediction as well as prognosis of gastric cancer (GC) in a Chinese population.

**Methods:** One thousand two hundred seventy-nine cases were enrolled, including 640 GC and 639 non-cancer cases. The functional tagSNPs of the TNFAIP2 gene were screened by Haploview software and NIH Snpinfo website. Human whole-blood genomic DNA was extracted by phenol chloroform method and analyzed by KASP SNP typing and sequencing method. ELISA was used to determine the expression of TNFAIP2 protein in serum samples. The miRNAs bound to TNFAIP2 3′ UTR rs8126 were predicted by MirSNP and TargetScan database. SPSS 22.0 software was used for statistical analysis, and *P* < 0.05 showed statistical difference.

**Results:** Four functional TNFAIP2 tagSNPs were found by bioinformatics analysis. TNFAIP2 rs8126 T>C polymorphism increased GC risk, and the risk in TC genotype cases was higher than that in TT genotype cases (*P* = 0.001, OR = 1.557). In the dominant model, the TNFAIP2 rs8126 polymorphic carrier was 1.419 times higher (*P* = 0.007). TNFAIP2 rs710100 C>T polymorphism, TNFAIP2 rs3759571 G>A polymorphism, and TNFAIP2 rs3759573 A>G polymorphism were not correlated with GC risk. In the subgroup analysis, TNFAIP2 rs8126 TC genotype cases had a higher GC risk in male, aged 60 years or older, *Helicobacter pylori*-negative, non-smoking, and non-drinking. However, there was no correlation between TNFAIP2 SNPs and GC prognosis. The TNFAIP2 protein concentration in GC patients was significantly different from that in healthy persons (*P* = 0.029), but it was not associated with GC prognosis. The high or low expression of TNFAIP2 protein had no significant difference with gender, age, *H. pylori* infection, smoking, and drinking in GC patients. The serum TNFAIP2 protein expression in rs8126 TT genotype carriers was significantly higher than that in rs8126 CC genotype carriers (*P* < 0.001).

**Conclusion:** TNFAIP2 3′ UTR rs8126 T>C polymorphism was associated with GC risk in a Chinese population, especially in cases with males aged 60 years or older, *H. pylori* negative, non-smoking and non-drinking. Compared with healthy persons, serum TNFAIP2 protein expression was higher in Chinese GC patients, and TNFAIP2 3′ UTR rs8126 T>C polymorphism might affect TNFAIP2 protein expression.

## Introduction

Gastric cancer (GC) is considered to be one of the most common malignant tumors in the world (1). It is usually asymptomatic or has mild symptoms in the early days but is prone to recurrence and metastasis due to tumor specificity and heterogeneity (2–4). In China, GC has become the second leading cause of cancer-related death, and the situation of disease prevention is extremely grim (5–7). So far, the pathogenesis of GC has not been completely clarified. Many etiological studies have found that some factors are closely related to GC, including environment, diet, microorganism, family inheritance, and physicochemical and genetic changes, especially specific oncogenes and tumor suppressor genes (8–10). In recent years, the Human Genome Atlas Project has provided a theoretical basis for exploring the correlation between genetic changes and malignant tumors. In nature, gene polymorphism is one of the most common forms of gene changes, and it can reflect the differences of biological activity between different individuals (11). The studies on gene polymorphism can lay an important foundation of molecular biology for revealing the mechanism of malignant tumors, and they have important roles in clarifying tumor susceptibility and predicting the development trend of tumors. Single nucleotide polymorphism (SNP), as the most common type of human genetic variation, is an important part of the research on gene polymorphism and can be used to explore the mechanism of tumor generation (12, 13).

Tumor necrosis factor alpha-induced protein 2 (TNFAIP2), also known as B94 and EXOC3L3, is a member of tumor necrosis factor alpha-induced proteins (TNFAIPs). It is located on human chromosome 14q32.32 and contains 14 exons, which has a genomic DNA span of 13.45 kDa and can encode a protein with 654 amino acids and a molecular weight of 72.6 kDa. TNFAIP2 interacts with EXOC1, EXOC2, EXOC4, EXOC7, and EXOC8 and participates in the formation and the development of human organs (14). It may also be involved in various biological processes such as angiogenesis, cell differentiation, bone marrow tissue generation, and spermatogenesis, and its main function is to regulate inflammation and angiogenesis (15). In *in vitro* studies, TNFAIP2 is believed to have differential expression during angiogenesis (16). In addition, TNFAIP2 also regulates the apoptosis of tumor cells and is considered to be a target gene for retinoic acid in acute promyelocytic leukemia (17). Previous studies have reported that functional TNFAIP2 SNPs, mainly located in the 3′ non-coding region (3′ UTR), may regulate gene expression by modifying the binding ability of miRNA to target genes and eventually lead to the differences in disease susceptibility. Recently, some studies have confirmed the relationship between TNFAIP2 SNPs and malignant tumors such as head and neck squamous cell carcinoma (SCCHN) and esophageal squamous cell carcinoma (ESCC), which is beneficial for screening high-risk groups and predicting outcomes of tumors (14, 15, 18, 19).

However, the correlation between TNFAIP2 gene polymorphism and prediction or prognosis of GC is rarely reported, especially in Asian or Chinese populations. At present, only one study from an American population reported that, compared with TT + TC genotype, the TNFAIP2 3′ UTR rs8126 CC genotype significantly increased GC risk, especially in the drinking population (14).

This study aims to understand the correlation between TNFAIP2 gene polymorphism and prediction or prognosis of GC in a Chinese population, explore the effect of TNFAIP2 gene polymorphism on the expression of TNFAIP2 protein, and attempt to provide a theoretical basis for molecular target prediction, disease diagnosis, and individualized treatment of GC.

## Materials and Methods

### Study Participants

**Figure 1:**
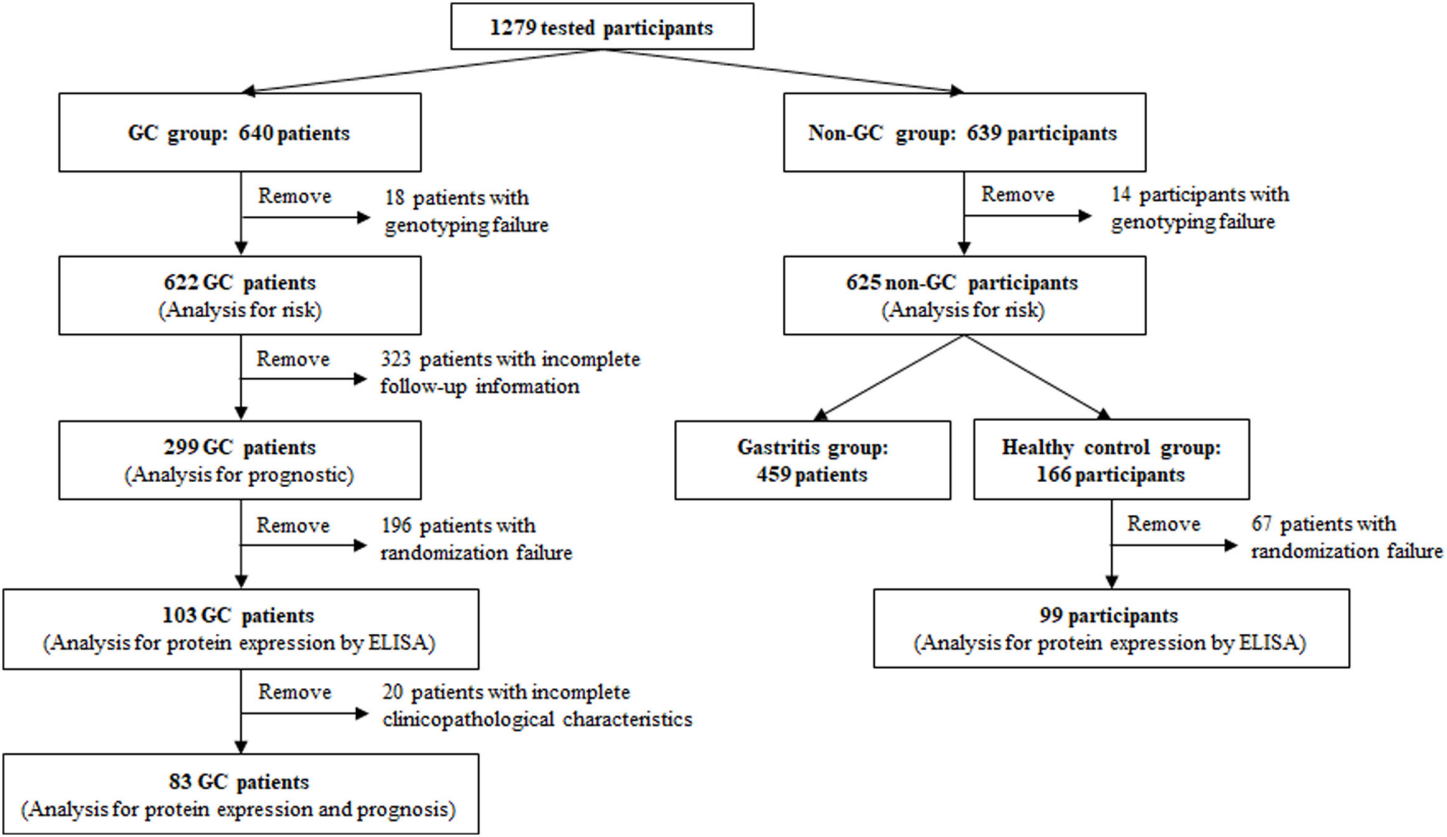
Participants' disposition. Human whole-blood genomic DNA tests were performed on 1,279 participants in this study, including 640 gastric cancer (GC) patients and 639 non-GC participants. Due to genotyping failure on some participants, the analysis of correlation between TNFAIP2 TagSNPs and GC risk was performed on 1,247 eligible participants, including 622 GC patients and 625 non-GC participants. Due to incomplete follow-up information, the analysis of correlation between TNFAIP2 TagSNPs and GC prognosis was performed on 299 GC patients. The analysis of TNFAIP2 protein expression and GC risk and prognosis was performed on 202 participants randomly selected from the GC group and the healthy control group, including 103 GC patients and 99 healthy persons. Due to incomplete clinicopathological characteristics, only 83 GC patients were enrolled in the analysis of correlation between serum TNFAIP2 protein expression and GC prognosis.

This was a case–control study from multiple medical centers in Liaoning Province, northern China, and 640 patients with GC and 639 non-GC cases were enrolled between December 1997 and December 2013. The inclusion criteria included the following: all participants had a clear pathological diagnosis and typing by electronic gastroscopy. The exclusion criteria included the following: (A) The participants had a major organ dysfunction; (B) The participants had autoimmune diseases; (C) The participants had other malignant tumors; and (D) The participants had infectious diseases. The fasting venous blood and serum of all participants were isolated and saved under the condition of 20°C below zero. The epidemiological information and the clinicopathological parameters of the cases were recorded, and the GC patients were followed up by telephone every 6 months. The main follow-up contents were overall survival, and the deadline for data collection was June 30, 2017 (Figure 1). This study was approved by the ethics committee of the First Affiliated Hospital of China Medical University [No. (2015)77], and all participants had signed the informed consent.

### Functional TagSNP Selection

The functional tagSNPs of the TNFAIP2 gene were screened by Haploview software and NIH Snpinfo website (https://snpinfo.niehs.nih.gov/). The F-SNP website (http://compbio.cs.queensu.ca/F-SNP/) and the NIH Snpinfo website were used to predict the functional tagSNPs, respectively. The parameters were set as: Chinese Han population, minimum allele frequency >5%, and frequency distribution *r*^2^ > 0.8 (Supplementary Figures 1, 2).

### Genotyping

Human whole-blood genomic DNA was extracted by phenol chloroform method and analyzed by KASP SNP typing and sequencing method. In the Sequenom MassARRAY platform (Sequenom, San Diego, CA, USA), SNP genotyping was performed by Bio Miao Biological Technology (Beijing, China). In addition, we randomly selected 10% of the samples for repeated analysis and found that the consistency rate of all the duplicated samples was 100%.

### Detection of Serum TNFAIP2 Protein and *H. pylori*-IgG by ELISA

Enzyme-linked immunosorbent assay (ELISA) was used to determine the expression of the TNFAIP2 protein in the serum samples. Double-antibody sandwich method was used for ELISA, and the ELISA kit was purchased from Shanghai Enzyme-linked Biotechnology Co., Ltd. The absorbance (OD value) was measured by Multiskan Ascent (Thermo Labsystems, USA) at 450 nm, and the TNFAIP2 concentration was calculated by a standard curve. Serum *H. pylori*-IgG titer was also detected by ELISA (*Helicobacter pylori* IgG kit; Biohit, Helsinki, Finland), and the details were described in our published study (20).

### Statistical Analysis

SPSS 20.0 software (SPSS Inc., Chicago, IL, USA) was used for statistical analysis. Firstly, we tested the normal distribution for units of measurement. If it conformed to the normal distribution, *T*-test could be used for statistical analysis. If it did not conform to the normal distribution, non-parametric test should be used for statistical analysis. The counting units were statistically analyzed by chi-square test. Multivariate logistic regression model was used to compare TNFAIP2 SNPs genotypes between the GC group and the non-GC group, and OR value and confidence interval (95% CI) were calculated to represent the relative risk. Logistic regression model was used to evaluate the interaction relationship between TNFAIP2 SNPs and *H. pylori* infection, smoking, and drinking. Adjusting for gender and age, a full-factor model was used to calculate the *P*-value of the interaction relationship between TNFAIP2 SNPs genotypes and *H. pylori* infection, smoking, and drinking. Cox proportional risk model was used for univariate and multivariate analysis to calculate the relationship between the clinical parameters and the prognosis of GC patients. *P* < 0.05 was considered as statistically significant.

## Results

### The Basic Characteristics of Study Participants

In this study, 1,247 qualified peripheral blood samples were analyzed for gene polymorphism, including 622 cases in the GC group and 625 cases in the non-GC group. Age and sex were matched in both groups. The mean age in the GC group and in the non-GC group was 59.26 ± 11.4 (26–87) and 58.53 ± 8.17 (26–89), respectively. The difference in *H. pylori* infection between the two groups was statistically significant (*P* < 0.001), but there were no significant differences in smoking and drinking (Table 1).

**Table 1:** The basic characteristics of the study participants.

**Basic characteristics**	**Gastric cancer (*n*, %)**	**Control (*n*, %)**	***P*-value**
Gender	*n* = 622	*n* = 625	0.381
Male	443 (71.2)	459 (73.4)	
Female	179 (28.8)	166 (26.6)	
Age (years)	*n* = 622	*n* = 625	0.195
Mean ± SD	59.26 ± 11.40	58.53 ± 8.17	
Median	59	58	
Range	26–87	26–89	
*H. pylori* infection^*^	*n* = 622	*n* = 625	**<0.001**
Positive	314 (50.5)	106 (17.0)	
Negative	308 (49.5)	519 (83.0)	
Smoking	*n* = 247	*n* = 361	0.359
Yes	98 (39.7)	130 (36.0)	
No	149 (60.3)	231 (64.0)	
Drinking	*n* = 247	*n* = 359	0.058
Yes	80 (32.4)	91 (25.3)	
No	167 (67.6)	268 (74.7)	

* *SPSS 20.0 random number generator was used to supplement the H. pylori infection status of 122 cases, whose H. pylori was unknown, so as to facilitate the subsequent statistical analysis. Bold Value indicate the data is statistically significant differences (P < 0.05)*.

### Functional TagSNPs Selected

Haploview software and NIH Snpinfo website were used to screen for functional tagSNPs, respectively. We found four functional TNFAIP2 SNPs and used them as candidate SNPs for further genotyping and statistical analysis, including miRNA binding sites (rs8126 and rs710100) and transcription factor binding sites (rs3759571 and rs3759573).

### The Correlation Between TNFAIP2 TagSNPs and GC Risk in General Population

A total of 1,247 samples were included to analyze the correlation between TNFAIP2 SNPs and GC risk. The wild and the mutant bases of SNPs were defined by searching the NCBI website. TNFAIP2 SNPs were classified by KASP SNP typing and sequencing as follows: wild type, heterozygous type, mutant type, dominant model, and recessive model. The differences of TNFAIP2 SNPs between the GC group and the non-GC group were compared, and the correlation between TNFAIP2 SNPs and GC risk was analyzed. The results showed that TNFAIP2 rs8126 T>C polymorphism was associated with GC risk in general populations, and the risk in TC genotype cases was higher than that in TT genotype cases (*P* = 0.001, OR = 1.557). In the dominant model, the GC risk in TNFAIP2 rs8126 polymorphic carriers was 1.419 times higher (*P* = 0.007). However, TNFAIP2 rs710100 C>T polymorphism, TNFAIP2 rs3759571 G>A polymorphism, and TNFAIP2 rs3759573 A>G polymorphism were not associated with GC risk. In particular, TNFAIP2 rs3759573 A>G polymorphism was not consistent with Hardy–Weinberg's genetic linkage balance (*P*_HWE_ < 0.05) and was excluded in the subsequent analysis (Table 2).

**Table 2:** The correlation between TNFAIP2 TagSNPs and gastric cancer (GC) risk in the general population.

**TNFAIP2 SNPs**	**GC (%)**	**Control (%)**	*P*-value^*^	OR^*^ (95% CI)
rs8126	*n* = 1125			
	*n* = 587	*n* = 538		
TT	272 (46.4)	205 (38.1)		1 (Ref)
TC	235 (40.0)	270 (50.2)	**0.001**	**1.557 (1.188–2.041)**
CC	80 (13.6)	63 (11.7)	0.901	1.026 (0.685–1.536)
CC + TC vs. TT			**0.007**	**1.419 (1.099–1.832)**
CC vs. TC + TT			0.298	0.818 (0.561–1.194)
*P*_HWE_		0.067		
rs710100	*n* = 1115			
	*n* = 543	*n* = 572		
CC	217 (40.0)	214 (37.4)		1 (Ref)
CT	251 (46.2)	285 (49.8)	0.545	0.920 (0.701–1.206)
TT	75 (13.8)	73 (12.8)	0.545	1.131 (0.156–0.332)
TT + CT vs. CC			0.805	0.968 (0.747–1.254)
TT vs. CT + CC			0.329	1.202 (0.831–1.738)
*P*_HWE_		0.145		
rs3759571				
	*n* = 578	*n* = 584		
GG	239 (41.3)	230 (39.4)		1 (Ref)
GA	268 (46.4)	278 (47.6)	0.597	0.931 (0.715–1.213)
AA	71 (12.3)	76 (13.0)	0.926	0.981 (0.662–1.455)
AA + GA vs. GG			0.672	0.947 (0.736–1.218)
AA vs. GA + GG			0.882	1.028 (0.711–1.488)
*P*_HWE_		0.575		
rs3759573				
	*n* = 529	*n* = 554		
AA	179 (33.8)	184 (33.2)		1 (Ref)
AG	291 (55.0)	302 (54.5)	0.858	1.026 (0.774–1.361)
GG	59 (11.2)	68 (12.3)	0.778	0.941 (0.614–1.440)
GG + AG vs. AA			0.918	1.014 (0.773–1.331)
GG vs. AG + AA			0.766	0.942 (0.633–1.400)
*P*_HWE_		**0.001^#^**		

* *Adjusted for gender, age, and H. pylori infection*.

# *The results were inconsistent with Hardy–Weinberg genetic linkage equilibrium. Bold Values indicate the data is statistically significant differences (P < 0.05)*.

### The Correlation Between TNFAIP2 TagSNPs and GC Risk in Subgroup Population

In the subgroup analysis, we found that, in male subjects, TNFAIP2 rs8126 TC genotype cases were associated with a higher GC risk than TT genotype cases (*P* = 0.005, OR = 1.573), and GC risk was 1.443 times higher in TNFAIP2 rs8126 polymorphic carriers in the dominant model (*P* = 0.018). In subjects aged over 60 years, TNFAIP2 rs8126 TC genotype cases had a higher GC risk than TT genotype cases (*P* = 0.005, OR = 1.816), and GC risk was 1.693 times higher in TNFAIP2 rs8126 polymorphic carriers in the dominant model (*P* = 0.010). In subjects younger than 60 years old, TNFAIP2 rs8126 TC genotype cases had a higher GC risk than TT genotype cases (*P* = 0.049, OR = 1.440). In subjects without *H. pylori* infection, TNFAIP2 rs8126 TC genotype cases had a higher GC risk than TT genotype cases (*P* = 0.006, OR = 1.560), and GC risk was 1.440 times higher in TNFAIP2 rs8126 polymorphic carriers in the dominant model (*P* = 0.017). In non-smoking subjects, TNFAIP2 rs8126 TC genotype cases had a higher GC risk than TT genotype cases (*P* = 0.038, OR = 1.701), and GC risk was 1.643 times higher in TNFAIP2 rs8126 polymorphic carriers in the dominant model (*P* = 0.038). In non-drinking subjects, TNFAIP2 rs8126 TC genotype cases had a higher GC risk than TT genotype cases (*P* = 0.045, OR = 1.630) (Table 3).

**Table 3:** The correlation between TNFAIP2 TagSNPs and gastric cancer (GC) risk in the subgroup population.

**Parameters**	**Genotype**	**GC vs. control**	*P*-value^*^	**OR (95%)**
rs8126				
Gender^#^		*n* = 587 vs. 538		
Male	TT	195/149		
	TC	171/201	**0.005**	**1.573 (1.143–2.164)**
	CC	55/45	0.841	1.051 (0.648–1.703)
	CC + TC vs. TT		**0.018**	**1.443 1.066**–**(1.954)**
	CC vs. TC + TT		0.407	0.825 (0.524–1.300)
Female	TT	77/56		
	TC	64/69	0.116	1.510 (0.903–2.525)
	CC	25/18	0.866	1.067 (0.500–2.275)
	CC + TC vs. TT		0.193	1.374 (0.852–2.216)
	CC vs. TC + TT		0.642	0.849 (0.425–1.694)
Age (years)		*n* = 587 vs. 538		
≥60	TT	129/74		
	TC	126/124	**0.005**	**1.816** (**1.195**–**2.758)**
	CC	34/25	0.493	1.257 (0.653–2.420)
	CC + TC vs. TT		**0.010**	**1.693** (**1.135**–**2.526)**
	CC vs. TC + TT		0.718	0.895 (0.488–1.638)
<60	TT	143/131		
	TC	109/146	**0.049**	**1.440** (**1.002**–**2.069)**
	CC	46/38	0.788	0.931 (0.551–1.572)
	CC + TC vs. TT		0.138	1.292 (0.921–1.811)
	CC vs. TC + TT		0.321	0.780 (0.477–1.274)
*H. pylori* infection^#^		*n* = 587 vs. 538		
Positive	TT	137/35		
	TC	121/46	0.084	1.569 (0.941–2.618)
	CC	41/9	0.757	0.879 (0.386–1.997)
	CC + TC vs. TT		0.186	1.391 (0.853–2.266)
	CC vs. TC + TT		0.361	0.698 (0.322–1.511)
Negative	TT	135/170		
	TC	114/224	**0.006**	**1.560** (**1.133**–**2.147)**
	CC	39/54	0.693	1.099 (0.687–1.759)
	CC + TC vs. TT		**0.017**	**1.440** (**1.067**–**1.944)**
	CC vs. TC + TT		0.563	0.878 (0.564–1.365)
Smoking		*n* = 246 vs. 314		
Yes	TT	47/44		
	TC	34/62	0.182	1.556 (0.813–2.979)
	CC	16/10	0.615	0.770 (0.277–2.135)
	CC + TC vs. TT		0.377	1.318 (0.715–2.432)
	CC vs. TC + TT		0.232	0.560 (0.216–1.450)
No	TT	76/74		
	TC	56/99	**0.038**	**1.701** (**1.030**–**2.809)**
	CC	17/25	0.298	1.501 (0.699–3.227)
	CC + TC vs. TT		**0.038**	**1.643** (**1.027**–**2.627)**
	CC vs. TC + TT		0.750	1.123 (0.549–2.298)
Drinking		*n* = 246 vs. 311		
Yes	TT	39/30		
	TC	29/43	0.089	1.831 (0.913–3.674)
	CC	12/6	0.579	0.718 (0.222–2.317)
	CC + TC vs. TT		0.216	1.518 (0.784–2.940)
	CC vs. TC + TT		0.233	0.515 (0.174–1.531)
No	TT	84/87		
	TC	61/117	**0.045**	**1.630** (**1.010**–**2.629)**
	CC	21/28	0.524	1.258 (0.620–2.552)
	CC + TC vs. TT		0.065	1.524 (0.974–2.384)
	CC vs. TC + TT		0.873	0.947 (0.485–1.851)
rs710100		*n* = 543 vs. 572		
Gender^#^				
Male	CC	151/166		
	CT	182/209	0.913	0.982 (0.713–1.352)
	TT	49/52	0.649	1.119 (0.689–1.816)
	TT + CT vs. CC		0.950	1.010 (0.744–1.371)
	TT vs. CT + CC		0.567	1.140 (0.728–1.787)
Female	CC	66/48		
	CT	69/76	0.251	0.738 (0.440–1.239)
	TT	26/21	0.877	1.060 (0.505–2.228)
	TT + CT vs. CC		0.427	0.818 (0.499–1.342)
	TT vs. CT + CC		0.439	1.298 (0.670–2.512)
Age (years)		*n* = 543 vs. 572		
≥60	CC	106/78		
	CT	131/131	0.373	0.827 (0.544–1.257)
	TT	33/24	0.461	1.290 (0.656–2.536)
	TT + CT vs. CC		0.581	0.892 (0.594–1.339)
	TT vs. CT + CC		0.274	1.410 (0.761–2.612)
<60	CC	111/136		
	CT	120/154	0.860	0.968 (0.673–1.391)
	TT	42/49	0.787	1.074 (0.641–1.800)
	TT + CT vs. CC		0.999	1.000 (0.710–1.409)
	TT vs. CT + CC		0.608	1.131 (0.706–1.812)
*H. pylori* infection^#^		*n* = 543 vs. 572		
Positive	CC	112/47		
	CT	124/44	0.536	1.168 (0.714–1.910)
	TT	36/7	0.080	2.227 (0.908–5.462)
	TT + CT vs. CC		0.258	1.313 (0.819–2.104)
	TT vs. CT + CC		0.104	2.031 (0.865–4.768)
Negative	CC	105/167		
	CT	127/241	0.272	0.833 (0.601–1.155)
	TT	39/66	0.676	0.905 (0.566–1.446)
	TT + CT vs. CC		0.313	0.853 (0.625–1.162)
	TT vs. CT + CC		0.945	1.015 (0.661–1.560)
Smoking		*n* = 228 vs. 337		
Yes	CC	37/48		
	CT	40/66	0.451	0.785 (0.418–1.474)
	TT	13/10	0.387	1.619 (0.543–4.823)
	TT + CT vs. CC		0.732	0.899 (0.490–1.651)
	TT vs. CT + CC		0.179	1.944 (0.737–5.125)
No	CC	61/82		
	CT	60/101	0.851	1.049 (0.635–1.735)
	TT	17/30	0.914	1.042 (0.492–2.210)
	TT + CT vs. CC		0.840	1.050 (0.652–1.693)
	TT vs. CT + CC		0.974	1.011 (0.505–2.025)
Drinking		*n* = 228 vs. 335		
Yes	CC	30/35		
	CT	34/46	0.570	0.820 (0.413–1.626)
	TT	10/5	0.354	1.826 (0.511–6.529)
	TT + CT vs. CC		0.825	0.928 (0.478–1.802)
	TT vs. CT + CC		0.178	2.238 (0.693–7.226)
No	CC	68/94		
	CT	66/120	0.947	0.984 (0.611–1.585)
	TT	20/35	0.892	1.050 (0.519–2.125)
	TT + CT vs. CC		0.965	1.010 (0.641–1.591)
	TT vs. CT + CC		0.879	1.052 (0.549–2.014)
rs3759571				
Gender^#^		*n* = 578 vs. 584		
Male	GG	163/172		
	GA	201/201	0.751	1.052 (0.769–1.438)
	AA	47/56	0.844	0.953 (0.592–1.534)
	AA + GA vs. GG		0.822	1.035 (0.768–1.395)
	AA vs. GA + GG		0.778	0.938 (0.601–1.463)
Female	GG	76/58		
	GA	67/77	0.128	0.678 (0.411–1.119)
	AA	24/20	0.848	0.930 (0.446–1.941)
	AA + GA vs. GG		0.218	0.743 (0.462–1.193)
	AA vs. GA + GG		0.620	1.188 (0.601–2.349)
Age (years)		*n* = 578 vs. 584		
≥60	GG	113/86		
	GA	141/121	0.408	0.841 (0.557–1.268)
	AA	28/31	0.353	0.735 (0.385–1.406)
	AA + GA vs. GG		0.324	0.819 (0.551–1.218)
	AA vs. GA + GG		0.528	0.823 (0.449–1.507)
<60	GG	126/144		
	GA	127/157	0.771	0.949 (0.667–1.349)
	AA	43/45	0.663	1.122 (0.668–1.884)
	AA + GA vs. GG		0.966	0.993 (0.712–1.385)
	AA vs. GA + GG		0.491	1.183 (0.733–1.907)
*H. pylori* infection^#^		*n* = 578 vs. 584		
Positive	GG	119/46		
	GA	140/44	0.510	1.178 (0.723–1.919)
	AA	34/8	0.249	1.656 (0.703–3.903)
	AA + GA vs. GG		0.338	1.256 (0.788–2.003)
	AA vs. GA + GG		0.306	1.530 (0.678–3.451)
Negative	GG	120/184		
	GA	128/234	0.279	0.840 (0.613–1.152)
	AA	37/68	0.425	0.828 (0.521–1.317)
	AA + GA vs. GG		0.253	0.840 (0.623–1.132)
	AA vs. GA + GG		0.676	0.912 (0.593–1.403)
Smoking		*n* = 236 vs. 350		
Yes	GG	42/50		
	GA	41/62	0.659	0.869 (0.465–1.624)
	AA	14/15	0.730	1.183 (0.456–3.070)
	AA + GA vs. GG		0.803	0.927 (0.511–1.680)
	AA vs. GA + GG		0.625	1.243 (0.519–2.978)
No	GG	62/88		
	GA	63/107	0.746	0.922 (0.565–1.506)
	AA	14/28	0.564	0.798 (0.371–1.716)
	AA + GA vs. GG		0.666	0.902 (0.565–1.440)
	AA vs. GA + GG		0.706	0.867 (0.413–1.819)
Drinking		*n* = 236 vs. 350		
Yes	GG	29/38		
	GA	38/46	0.736	1.125 (0.568–2.227)
	AA	10/5	0.200	2.225 (0.655–7.561)
	AA + GA vs. GG		0.535	1.230 (0.640–2.365)
	AA vs. GA + GG		0.236	2.039 (0.628–6.625)
No	GG	75/100		
	GA	66/121	0.261	0.765 (0.480–1.220)
	AA	18/38	0.244	0.664 (0.334–1.321)
	AA + GA vs. GG		0.194	0.746 (0.479–1.161)
	AA vs. GA + GG		0.481	0.788 (0.407–1.527)

* *Adjusted for gender, age, and H. pylori infection*.

# *Adjusted for two other factors besides self. Bold Values indicate the data is statistically significant differences (P < 0.05)*.

### The Interaction Effects Between TNFAIP2 TagSNPs and Environmental Factors on GC Risk

The interaction effects between TNFAIP2 SNPs (rs8126, rs710100, and rs3759571) and environmental factors (*H. pylori* infection, smoking, and drinking) on GC risk were analyzed, and the results showed that there was no significant correlation between them (*P*_interaction_ > 0.05; Table 4).

**Table 4:** The interaction effects between TNFAIP2 TagSNPs and environmental factors on gastric cancer (GC) risk.

**SNP genotype**	***H. pylori*** **infection**		**Smoking**		**Drinking**	
	**Positive**	**Negative**	**Yes**	**No**	**Yes**	**No**
rs8126	*n* = 389	*n* = 736	*n* = 213	*n* = 347	*n* = 159	*n* = 398
**TT**						
GC/control (CON)	137/35	135/170	47/44	76/74	39/30	84/87
OR (95% CI)	4.858 (3.527–6.692)	1 (Ref)	0.338 (0.201–0.567)	1 (Ref)	0.282(0.170–0.468)	1 (Ref)
**TC** **+** **CC**						
GC/CON	162/55	153/278	50/72	72/127	41/49	82/145
OR (95% CI)	2.975(1.807–4.898)	0.432(0.293–0.635)	0.412(0.211–0.805)	1.012(0.683–1.501)	0.729(0.362–1.471)	1.144(0.750–1.747)
	*P*_interaction_ = 0.788		*P*_interaction_ = 0.793		*P*_interaction_ = 0.823	
	OR = 0.925 (0.524–1.632)		OR = 0.910 (0.451–1.836)		OR = 0.918(0.432–1.950)	
rs710100	*n* = 370	*n* = 745	*n* = 214	*n* = 351	*n* = 160	*n* = 403
**CC**						
GC/CON	112/47	105/167	37/48	61/82	30/35	68/94
OR (95% CI)	3.790 (2.493–5.763)	1 (Ref)	1.036 (0.603–1.782)	1 (Ref)	1.185(0.664–2.114)	1 (Ref)
**TC** **+** **TT**		
GC/CON	160/51	166/307	53/76	77/131	44/51	86/155
OR (95% CI)	4.990 (3.349–7.434)	0.860 (0.632–1.171)	0.937 (0.579–1.519)	0.790 (0.512–1.220)	1.193(0.716–1.986)	0.767(0.510–1.154)
	*P*_interaction_ = 0.119		*P*_interaction_ = 0.827		*P*_interaction_ = 0.604	
	OR = 1.560 (0.892–2.728)		OR = 1.082 (0.532–2.201)		OR = 1.222 (0.572–2.612)	
rs3759571	*n* = 391	*n* = 771	*n* = 224	*n* = 362	*n* = 166	*n* = 418
**GG**						
GC/CON	119/46	120/184	42/50	62/88	29/38	75/100
OR (95% CI)	3.967 (2.631–5.981)	1 (Ref)	1.192 (0.706–2.012)	1 (Ref)	1.018(0.576–1.797)	1(Ref)
**GA** **+** **AA**						
GC/CON	174/52	165/302	55/77	77/135	48/51	84/159
OR (95% CI)	5.131 (3.488–7.546)	0.838 (0.622–1.129)	1.014 (0.631–1.630)	0.810 (0.527–1.243)	1.225(0.765–2.059)	0.704(0.472–1.050)
	*P*_interaction_ = 0.123		*P*_interaction_ = 0.944		*P*_interaction_ = 0.156	
	OR = 1.540 (0.890–2.666)		OR = 1.025 (0.513–2.048)		OR = 1.715 (0.815–3.610)	

### The Correlation Between TNFAIP2 TagSNPs and GC Prognosis

Prognostic analysis was performed in 299 GC patients who had complete survival follow-up data. We found that GC prognosis was correlated with Borrmann classification, depth of invasion, growth pattern, lymphatic vessel invasion, lymph node metastasis, and TNM stage (Table 5). Both univariate analysis and multivariate analysis showed no statistical differences between TNFAIP2 SNPs and GC prognosis (*P* > 0.05), suggesting that TNFAIP2 SNPs had nothing to do with GC prognosis in this group (Table 6). In the subgroup analysis, TNFAIP2 rs8126 polymorphism was stratified by gender, age, and *H. pylori* infection, and no correlation was found between TNFAIP2 rs8126 polymorphism and GC prognosis (*P* > 0.05) (Table 7).

**Table 5:** The correlation between basic characteristics and gastric cancer (GC) prognosis.

**Basic**	**GC patients**	**Death**	**Median survival**	***P*-value**
**characteristics**			**time (mean)**	
Total	*n* = 299	*n* = 124		
Gender				0.097
Male	219	92	79.0^a^	
Female	80	32	54.1^b^	
Age (years)				0.553
≥60	141	61	58.0^a^	
<60	158	63	79.0^a^	
*H. pylori* infection				0.334
Positive	157	61	56.7^b^	
Negative	142	63	58.0^a^	
Smoking				0.718
Yes	98	41	79.0^a^	
No	149	64	52.9^b^	
Drinking				0.703
Yes	80	35	79.0^a^	
No	167	70	53.6^b^	
Family history				0.570
Yes	33	13	68.0^a^	
No	210	93	79.0^a^	
Borrmann classification				**<0.001**
Borrmann I–II	69	22	64.8^b^	
Borrmann III–IV	199	98	47.0^a^	
Lauren classification				0.594
Intestinal type	109	43	56.2^b^	
Diffuse type	189	81	79.0^a^	
Site of primary lesions				
Corpus	81	34	52.0^b^	0.513
Fundus	31	9	64.1^b^	
Antrum/angle	123	54	79.0^a^	
Growth pattern				**0.035**
Infiltrative	136	67	40.0^a^	
Intermediate/expanding	106	35	61.8^b^	
Depth of invasion				**<0.001**
T1/T2	130	22	75.3^b^	
T3/T4	169	102	29.0^a^	
TNM stage				**0.001**
I–II	85	22	65.2^b^	
III–IV	214	102	57.0^a^	
Lymph node metastasis				**<0.001**
Positive	178	102	35.0^a^	
Negative	121	22	70.1^b^	
Lymphatic vessel invasion				**<0.001**
Positive	34	24	31.0^a^	
Negative	182	62	59.3^b^	
Blood vessel invasion				0.061
Positive	23	14	20.0^a^	
Negative	193	72	57.8^b^	

a *Median survival time*.

b *Mean survival time. Bold Values indicate the data is statistically significant differences (P < 0.05)*.

**Table 6:** The correlation between TNFAIP2 SNPs and gastric cancer (GC) prognosis in the general analysis.

**TNFAIP2 SNPs**	**GC**	**Death**	**Median survival time (mean)**	**Univariate analysis**		**Multivariate analysis**	
				***P*-value**	**HR (95% CI)**	*P*-value^*^	**HR (95% CI)**
rs8126	*n* = 287	*n* = 120					
TT	137	58	56.4^b^				
TC	109	44	79.0^a^	0.840	0.960 (0.649–1.421)	0.501	1.147 (0.770–1.707)
CC	41	18	68.0^a^	0.840	1.056 (0.622–1.792)	0.399	1.262 (0.735–2.165)
CC + TC vs. TT				0.932	1.008 (0.843–1.205)	0.408	1.166 (0.811–1.676)
CC vs. TC + TT				0.793	0.967 (0.753–1.242)	0.588	1.151 (0.692–1.915)
rs710100	*n* = 263	*n* = 111					
CC	110	49	68.0^a^				
TC	114	46	79.0^a^	0.468	1.161 (0.776–1.736)	0.349	0.824 (0.549–1.236)
TT	39	16	68.0^a^	0.513	1.099 (0.829–1.457)	0.638	0.871 (0.489–1.550)
TC + TT vs. CC				0.394	1.085 (0.899–1.309)	0.329	0.828 (0.567–1.209)
TT vs. CC + TC				0.643	1.065 (0.817–1.388)	0.713	0.904 (0.528–1.547)
rs3759571	*n* = 275	*n* = 113					
GG	113	45	58.2^b^				
GA	124	53	79.0^a^	0.685	0.921 (0.619–1.370)	0.803	0.950 (0.635–1.421)
AA	38	15	55.1^b^	0.951	1.009 (0.753–1.352)	0.325	0.739 (0.405–1.349)
GA + GG vs. AA				0.772	0.973 (0.806–1.174)	0.599	0.902 (0.614–1.324)
GG vs. GA + AA				0.780	1.039 (0.792–1.364)	0.335	0.762 (0.438–1.324)

* *Borrmann classification, TNM staging, lymph node metastasis, and depth of invasion were taken as covariables*.

a *Median survival time*.

b *Mean survival time*.

**Table 7:** The correlation between TNFAIP2 rs8126 polymorphism and gastric cancer (GC) prognosis in the subgroup analysis.

**Parameters**	**Genotype**	**GC**	**Death**	**Median survival time (mean)**	**Univariate analysis**		**Multivariate analysis**	
					***P*-value**	**HR (95% CI)**	*P*-value^*^	**HR (95% CI)**
rs8126		*n* = 287	*n* = 120					
Gender								
Male	TT	103	44	56.3^b^				
	TC	79	32	79.0^a^	0.843	0.955 (0.606–1.506)	0.488	1.177 (0.743–1.864)
	CC	29	13	68.0^a^	0.961	1.016 (0.547–1.886)	0.795	1.087 (0.579–2.039)
	CC + TC vs. TT				0.892	0.972 (0.641–1.472)	0.499	1.156 (0.760–1.758)
	CC vs. TC + TT				0.912	1.034 (0.574–1.862)	0.948	1.020 (0.562–1.850)
Female	TT	34	14	50.4^b^				
	TC	30	12	51.8^b^	0.943	1.029 (0.476–2.225)	0.762	1.132 (0.506–2.532)
	CC	12	5	54.3^b^	0.700	1.223 (0.439–3.405)	0.081	2.729 (0.883–8.431)
	CC + TC vs. TT				0.846	1.073 (0.529–2.177)	0.522	1.275 (0.606–2.679)
	CC vs. TC + TT				0.719	1.192 (0.457–3.112)	0.278	1.733 (0.641–4.681)
Age (years)		*n* = 287	*n* = 120					
≥60	TT	65	29	58.0^a^				
	TC	51	23	57.0^a^	0.925	1.027 (0.593–1.776)	0.506	1.210 (0.690–2.124)
	CC	20	7	58.9^b^	0.400	0.701 (0.307–1.603)	0.570	0.783 (0.336–1.823)
	CC + TC vs. TT				0.765	0.925 (0.555–1.543)	0.788	1.074 (0.638–1.809)
	CC vs. TC + TT				0.371	0.697 (0.317–1.536)	0.446	0.732 (0.329–1.632)
<60	TT	72	29	53.8^b^				
	TC	58	21	79.0^a^	0.673	0.886 (0.505–1.554)	0.968	1.012 (0.570–1.797)
	CC	21	11	68.0^a^	0.332	1.410 (0.704–2.826)	0.147	1.690 (0.832–3.435)
	CC + TC vs. TT				0.961	1.013 (0.612–1.674)	0.501	1.192 (0.715–1.985)
	CC vs. TC + TT				0.224	1.501 (0.780–2.888)	0.152	1.628 (0.836–3.170)
*H. pylori* infection		*n* = 287	*n* = 120					
Positive	TT	76	29	56.7^b^				
	TC	56	23	79.0^a^	0.660	1.131 (0.654–1.956)	0.108	1.583 (0.904–2.772)
	CC	20	6	63.1^b^	0.437	0.705 (0.292–1.700)	0.549	0.760 (0.309–1.865)
	CC + TC vs. TT				0.999	1.000 (0.597–1.673)	0.294	1.329 (0.781–2.261)
	CC vs. TC + TT				0.338	0.661 (0.284–1.542)	0.345	0.662 (0.282–1.557)
Negative	TT	61	29	58.0^a^				
	TC	53	21	54.1^b^	0.427	0.796 (0.454–1.397)	0.488	0.816 (0.460–1.450)
	CC	21	12	29.0^a^	0.361	1.369 (0.698–2.686)	0.101	1.792 (0.893–3.595)
	CC + TC vs. TT				0.779	0.931 (0.565–1.534)	0.902	0.969 (0.586–1.604)
	CC vs. TC + TT				0.196	1.516 (0.807–2.850)	0.080	1.794 (0.932–3.454)

* *Borrmann classification, TNM staging, lymph node metastasis, and depth of invasion were taken as covariables*.

a *Median survival time*.

b *Mean survival time*.

### Serum TNFAIP2 Protein Expression Between GC Patients and Healthy Persons

ELISA was performed on 202 serum samples randomly selected from the GC group and the healthy control group, including 103 GC patients and 99 healthy persons. There was no statistical difference in age, gender, and TNFAIP2 rs8126 genotypes between the two groups. The average age of the GC group and the healthy control group was 56.57 ± 7.656 (29–67) years old and 54.45 ± 7.737 (43–81) years old, respectively. The TNFAIP2 protein concentration in GC patients was significantly different from that in healthy persons (*P* = 0.029; Table 8).

**Table 8:** Serum TNFAIP2 protein expression between gastric cancer (GC) patients and healthy persons.

**Basic characteristics**	**GC (*n*, %)**	**Control (*n*, %)**	***P***
Total	*n* = 103	*n* = 99	
Gender			0.085
Male	78 (75.7)	64 (64.6)	
Female	25 (24.3)	35 (35.4)	
Age (years)			
Mean ± SD	56.57 ± 7.656	54.45 ± 7.737	0.052
Median	58	53	
Range	29–67	43–81	
TNFAIP2 concentration (ng/ml)			0.029^*^
Median (QR)	14.82 (19.56)	14.32 (2.85)	
Range	8.10–204.05	1.28–49.09	
TNFAIP2 rs8126 genotypes			0.941
TT	48 (46.6)	38 (38.4)	
TC	45 (43.7)	50 (50.5)	
CC	10 (9.7)	11 (11.1)	

* *Non-parametric test. Bold Value indicate the data is statistically significant differences (P < 0.05)*.

### The Correlation Between Serum TNFAIP2 Protein Expression and Clinicopathological Parameters in GC Patients

According to median TNFAIP2 protein concentration, 103 GC patients were divided into high-expression group and low-expression group, and the correlation between TNFAIP2 protein expression and clinicopathological parameters in GC patients was analyzed. We found that a high or a low expression of TNFAIP2 protein had no significant difference with gender, age, *H. pylori* infection, smoking, and drinking (Table 9).

**Table 9:** The correlation between serum TNFAIP2 protein expression and clinicopathological parameters in gastric cancer (GC) patients.

**Clinicopathological**	**TNFAIP2 protein expression**		***P***
**parameters**	**in GC patients**		
	**High expression**	**Low expression**	
	**concentration ≥**	**concentration <**	
	**14.82ng/ml (*n*, %)**	**14.82 ng/ml (*n*, %)**	
Total	*n* = 51	*n* = 52	
Gender	*n* = 51	*n* = 52	0.274
Male	41 (80.4)	37 (71.2)	
Female	10 (19.6)	15 (28.8)	
Age (years)	*n* = 51	*n* = 52	0.716
Mean ± SD	56.29 ± 8.008	56.85 ± 7.363	
Median	58	58	
Range	29–67	30–67	
*H. pylori* infection	*n* = 51	*n* = 52	0.754
Positive	21 (41.2)	23 (44.2)	
Negative	30 (58.8)	29 (55.8)	
Smoking	*n* = 42	*n* = 41	0.198
Yes	18 (42.9)	12 (29.3)	
No	24 (57.1)	29 (70.7)	
Drinking	*n* = 42	*n* = 41	0.261
Yes	15 (35.7)	10 (24.4)	
No	27 (64.3)	31 (75.6)	

### The Correlation Between Serum TNFAIP2 Protein Expression and GC Prognosis

A total of 83 cases with complete clinical data and survival data were selected from 103 GC patients. The basic characteristics of the patients included gender, age, *H. pylori* infection, smoking, drinking, family history, Borrmann classification, Lauren classification, site of primary lesions, growth pattern, depth of invasion, TNM stage, and lymph node metastasis. We found significant differences in depth of invasion (*P* < 0.001) and lymph node metastasis (*P* = 0.002; Table 10). According to serum TNFAIP2 protein concentration, the univariate analysis showed that TNFAIP2 protein expression was not significantly correlated with GC prognosis (*P* = 0.798; hazard ratio, HR = 1.090). The multivariate analysis with depth of invasion and lymph node metastasis as covariables confirmed that there was no significant difference in GC prognosis between the two groups (*P* = 0.339; HR = 1.387). The results suggested that serum TNFAIP2 protein expression was not associated with the prognosis of GC patients in this group (Table 11).

**Table 10:** The correlation between basic characteristics and survival in gastric cancer (GC) patients.

**Basic**	**GC patients**	**Death**	**Median survival**	***P*-value**
**characteristics**			**time (mean)**	
**Total**	***n*** **=** **35**	***n*** **=** **48**		
Gender				0.592
Male	28 (80.0)	36 (75.0)	40.8^b^	
Female	7 (20.0)	12 (25.0)	53.0^b^	
Age (years)				0.384
≥60	23 (65.7)	27 (56.2)	53.0^a^	
<60	12 (34.3)	21 (43.8)	46.0^b^	
*H. pylori* infection				0.328
Positive	13 (37.1)	23 (47.9)	42.4^b^	
Negative	22 (62.9)	25 (52.1)	30.0^a^	
Smoking				0.763
Yes	12 (34.3)	18 (37.5)	39.1^b^	
No	23 (65.7)	30 (62.5)	53.0^a^	
Drinking				0.793
Yes	10 (28.6)	15 (31.2)	39.2^b^	
No	25 (71.4)	33 (68.8)	53.0^a^	
Family history				1.000^*^
Yes	2 (5.7)	4 (8.3)	36.8^b^	
No	33 (94.3)	44 (91.7)	42.0^b^	
Borrmann classification				0.448^*^
Borrmann I–II	4 (11.4)	3 (6.2)	29.0^a^	
Borrmann III–IV	31 (88.6)	45 (93.8)	42.6^b^	
Lauren classification				0.719
Intestinal type	13 (37.1)	16 (33.3)	46.0^a^	
Diffuse type	22 (62.9)	32 (66.7)	39.3^b^	
Site of primary lesions				
Corpus	13 (37.1)	14 (29.2)	32.0^a^	0.189
Fundus	1 (2.9)	7 (14.6)	49.9^b^	
Antrum/angle	21 (60.0)	27 (56.2)	38.5^b^	
Growth pattern				0.621
Infiltrative	26 (81.2)	36 (76.6)	41.8^b^	
Intermediate/expanding	6 (18.8)	11 (23.4)	42.3^b^	
Depth of invasion				**<0.001**
T1/T2	3 (8.6)	24 (50.0)	53.7^b^	
T3/T4	32 (91.4)	24 (50.0)	24.0^a^	
TNM stage				0.456
I–II	7 (20.0)	13 (27.1)	42.8^b^	
III–IV	28 (80.0)	35 (72.9)	53.0^a^	
Lymph node metastasis				**0.002**
Positive	28 (80.0)	22 (45.8)	26.0^a^	
Negative	7 (20.0)	26 (54.2)	48.4^b^	

a *Median survival time*.

b *Mean survival time*.

* *Fisher's exact test. Bold Values indicate the data is statistically significant differences (P < 0.05)*.

**Table 11:** The correlation between serum TNFAIP2 protein expression and gastric cancer (GC) prognosis.

**TNFAIP2 protein concentration**	**GC**	**Death**	**Median survival time (mean)**	**Univariate analysis**		**Multivariate analysis**	
				***P*-value**	**HR (95% CI)**	P^*^	**HR (95% CI)**
	*n* = 83	*n* = 48		0.798	1.090 (0.562–2.116)	0.339	1.387 (0.710–2.710)
High expression concentration ≥ 14.82 ng/ml	42	24	53.0^a^				
Low expression concentration <14.82 ng/ml	41	24	43.0^b^				

* *Depth of invasion and lymph node metastasis were taken as covariables*.

a *Median survival time*.

b *Mean survival time*.

### The Correlation Between TNFAIP2 3′ UTR rs8126 T>C Polymorphism and TNFAIP2 Protein Expression

The correlation between TNFAIP2 3′ UTR rs8126 T>C polymorphism and TNFAIP2 protein expression was analyzed by different polymorphism genotypes in 103 GC patients, and we found that TNFAIP2 protein expression in rs8126 TT genotype carriers was significantly higher than that in rs8126 CC genotype carriers (*P* < 0.001) (Table 12).

**Table 12:** The correlation between TNFAIP2 3′ UTR rs8126 T > C polymorphism and TNFAIP2 protein expression.

**Basic characteristics**	**TNFAIP2 3**^**′**^ **UTR rs8126 T** **>** **C polymorphism**			***P***
	**TT**	**TC**	**CC**	
Total	*n* = 48	*n* = 45	*n* = 10	
TNFAIP2 protein concentration (ng/ml)^*^				<**0.001**
Median (QR)	22.72 (34.26)	13.06 (4.13)	13.24 (12.50)	
Range	8.10–204.05	9.10–142.9	10.48–48.11	

* *Nonparametric test. Bold Value indicate the data is statistically significant differences (P < 0.05)*.

## Discussion

TNFAIP2 is a novel gene induced by TNF-α and can regulate inflammatory and tumor angiogenesis (21). In recent years, studies have found that SNPs in mRNA 3′ UTR may impact the miRNA-mediated expression and regulation of oncogenes and tumor suppressors and confirmed that TNFAIP2 3′ UTR SNPs are correlated with risk of multiple malignancies, especially that TNFAIP2 3′ UTR rs8126 polymorphism may affect TNFAIP2 expression in GC, SCCHN, and ESCC by disturbing the binding of miR-184 with TNFAIP2 mRNA (14, 18, 19). However, only one study reports the correlation between TNFAIP2 SNPs and GC risk in the American population (14), and the correlation between TNFAIP2 SNPs and GC prognosis has not been reported until now, especially in Asian or Chinese populations.

This is the first study about TNFAIP2 SNPs in Chinese Han population, and this explored the correlation between TNFAIP2 SNPs and prediction as well as the prognosis of GC in a large sample population and its effect on TNFAIP2 protein expression. By analyzing TNFAIPS SNP genotyping of 1,247 samples, we found that the GC risk in TNFAIP2 rs8126 TC genotype cases was higher than that in TT genotype cases (*P* = 0.001, OR = 1.557), and the GC risk in polymorphic carriers of TNFAIP2 rs8126 was increased to 1.419 times in the dominant model (*P* = 0.007). These results were consistent with the American study and confirmed the correlation between TNFAIP2 rs8126 polymorphism and GC risk (14). In the subgroup analysis, we found that cases with TNFAIP2 rs8126 TC genotype had a higher GC risk in males, aged 60 years or older, *H. pylori* negative, non-smoking, and non-drinking. These results suggested that TNFAIP2 rs8126 T>C polymorphism was an important factor in predicting GC risk, and it is beneficial to the discovery and the diagnosis of early gastric cancer.

This study is the first to report the interaction effects between *H. pylori* infection and TNFAIP2 SNPs on GC risk. *H. pylori* infection is currently considered to be one of the environmental factors closely related to the risk and prognosis of GC (22, 23). Clarifying the interaction effects between TNFAIP2 SNPs and *H. pylori* infection is conducive to revealing the influence of key environmental factors on GC risk. Our results showed that there was no interaction between *H. pylori* infection and TNFAIP2 SNPs (rs8126, rs710100, and rs3759571) (*P*_interaction_ > 0.05), suggesting that the interaction effects between *H. pylori* infection and TNFAIP2 SNPs could not affect GC risk in this group, and no other similar results had been reported so far. In addition, we analyzed the interaction effects between smoking and drinking and TNFAIP2 SNPs on GC risk and found that there was no interaction between smoking and drinking and TNFAIP2 SNPs on GC risk (*P*_interaction_ > 0.05). This result was different from that of the American population (14), which may be related to differences in race, dietary habits and diet, and type and content of alcohol between Chinese and Americans.

This study also revealed the correlation between TNFAIP2 SNPs and GC prognosis in a Chinese population for the first time. Both univariate and multivariate analyses in the general population and in the subgroup suggested that TNFAIP2 rs8126 T>C polymorphism, TNFAIP2 rs3759571 G>A polymorphism, and TNFAIP2 rs3759573 A>G polymorphism were not related to GC prognosis. These results were not entirely consistent with those reported in other tumors. For example, TNFAIP2 was an independent prognostic factor for nasopharyngeal carcinoma (24) and TNFAIP2 3′ UTR rs8126 may shorten the survival time of patients with septic shock (16).

At the same time, the serum of 202 participants was tested by ELISA to explore differences in TNFAIP2 protein expression between GC patients and healthy persons. We found that the TNFAIP2 protein concentration in GC patients was significantly higher than that in healthy persons, suggesting that the TNFAIP2 protein may be more highly expressed in GC patients. However, the clinicopathological parameters such as gender, age, *H. pylori* infection, smoking, and drinking in GC patients did not affect serum TNFAIP2 protein expression. In addition, we analyzed the correlation between basic characteristics and survival in GC patients and found that GC patients with T1/T2 invasion depth and no lymph node metastasis had a better prognosis, but both the univariate analysis and the multivariate analysis showed that TNFAIP2 protein expression was not significantly correlated with GC prognosis, suggesting that serum TNFAIP2 protein expression was not associated with GC prognosis.

In the last part, we revealed the correlation between TNFAIP2 3′ UTR rs8126 T>C polymorphism and TNFAIP2 protein expression. As far as we know, 3′ UTR consisted of cis-/trans elements and may affect mRNA translation, stability, and subcellular localization. In malignant tumors, the reprogramming of 3′ UTRs mainly included cleavage, polyadenylation, chromosomal rearrangements, hormone-regulated 3′ UTR processing, point mutations, and polymorphisms (25). Therefore, abnormal gene expression caused by reprogramming nucleotides in 3'UTRs might be one of the important factors leading to the occurrence and the progression of tumors. rs8126 was located in the 3′ UTR of the TNFAIP2 gene sequence. A previous study showed that the rs8126 genetic variant was significantly associated with increased ESCC risk in a Chinese population (19). In this paper, our results showed that the serum TNFAIP2 protein expression in rs8126 TT genotype carriers was significantly higher than that in rs8126 CC genotype carriers, and it was suggested that TNFAIP2 3′ UTR rs8126 T>C polymorphism could affect serum TNFAIP2 protein expression. Our data also validated the previous hypothesis that functional genetic variants in 3′ UTR of gene might influence miRNA-mediated expression and regulation of mRNA.

As far as we know, this study has the largest sample size about TNFAIP2 SNPs in a Chinese Han population until now, and the study is the first to reveal the correlation between TNFAIP2 SNPs and GC risk, prognosis, and related risk factors in Chinese people. In addition, this is the first report on the correlation between serum TNFAIP2 protein expression and GC risk and prognosis. However, there are some limitations in this paper. For example, due to the lack of statistical data on previous treatment history, therapeutic effect, concomitant diseases, and other prognostic factors, these might affect the reliability of partial results, and the above results needed to be verified by further studies.

To sum up, TNFAIP2 3′ UTR rs8126 T>C polymorphism is associated with GC risk in a Chinese population, especially in cases with males, aged 60 years or older, *H. pylori*-negative, non-smoking, and non-drinking. However, there was no correlation between TNFAIP2 SNPs and GC prognosis. Compared with healthy persons, serum TNFAIP2 protein expression was higher in GC patients, but it was not associated with GC prognosis. In addition, TNFAIP2 3′ UTR rs8126 T>C polymorphism might affect serum TNFAIP2 protein expression, and the mechanism remains to be further explored.

## Data Availability Statement

The datasets presented in this study can be found in online repositories. The names of the repository/repositories and accession number(s) can be found below: dbSNP (https://www.ncbi.nlm.nih.gov/snp/—ss2137544092, ss3984446983, ss3984446984, and ss3984446985).

## Ethics Statement

The studies involving human participants were reviewed and approved by Medical Science Research Ethics Committee of the First Affiliated Hospital of China Medical University. The patients/participants provided their written informed consent to participate in this study.

## Author Contributions

YY and FG: conceived and designed the experiments. FG: performed the experiments. FG, QX, ZL, H-XD, Z-DZ, and L-PS: collected the samples and analyzed the data. YY: contributed reagents, materials, and analysis tools. FG and YY: wrote and revised the paper. All authors: read and approved the final manuscript.

## Conflict of Interest

The authors declare that the research was conducted in the absence of any commercial or financial relationships that could be construed as a potential conflict of interest.

## Supplementary Material

Supplementary Figure 1 Linkage disequilibrium diagram on TNFAIP2 tagSNPs by Haploview software. The tagSNPs of the TNFAIP2 gene were screened by Haploview software and F-SNP website was used to predict the function of tagSNPs. The parameters were set as Chinese Han population; minimum allele frequency >5%; frequency distribution *r*^2^ > 0.8. This linkage disequilibrium diagram showed that rs2234130, rs710100, rs146514706, and rs1132339 were tagSNPs of the TNFAIP2 gene, and the alleles of rs2234130 included rs8126, rs3759571, rs3759573, rs2234130, rs749206, rs4369588, rs2234143, rs8176365, rs2234131, rs2403128, rs944000, rs1887940, rs2234133, rs4283165, and rs11160713.

Supplementary Figure 2 Prediction diagram on TNFAIP2 tagSNPs by the NIH Snpinfo website. The functional tagSNPs of the TNFAIP2 gene were predicted by the NIH Snpinfo website. The parameters were set as Chinese Han population; minimum allele frequency >5%; frequency distribution *r*^2^ > 0.8. This prediction diagram showed that rs1887940 and rs710100 were tagSNPs of the TNFAIP2 gene, and the alleles of rs1887940 included rs8126, rs1887940, rs2234130, and rs749206.
