# Supplementary figures and images for: Correlation Between TNFAIP2 Gene Polymorphism and Prediction/Prognosis for Gastric Cancer and Its Effect on TNFAIP2 Protein Expression
Source: Front Oncol. 2020 Jul 24;10:1127. doi: 10.3389/fonc.2020.01127 (PMC7394262; doi:10.3389/fonc.2020.01127)

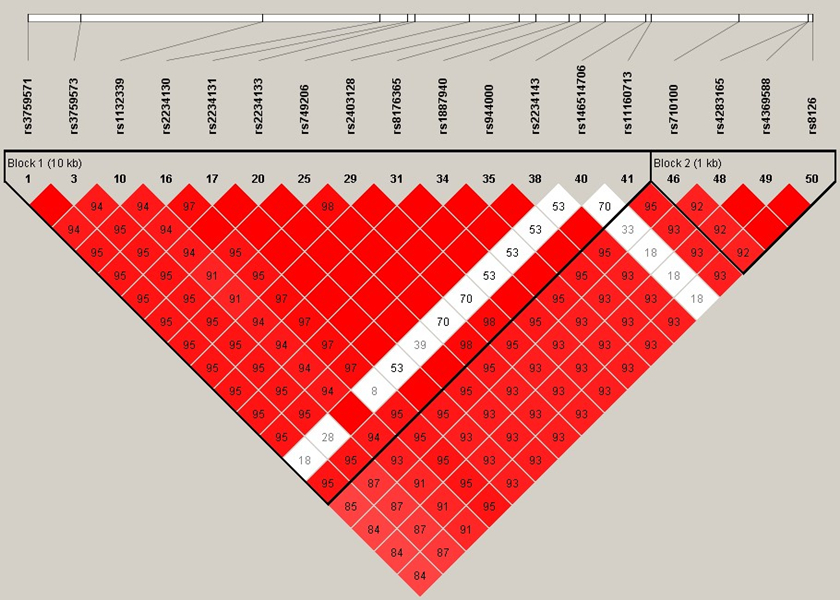

Supplement: Supplementary Figure 1 — Linkage disequilibrium diagram on TNFAIP2 tagSNPs by Haploview software. The tagSNPs of the TNFAIP2 gene were screened by Haploview software and F-SNP website was used to predict the function of tagSNPs. The parameters were set as Chinese Han population; minimum allele frequency >5%; frequency distribution r2 > 0.8. This linkage disequilibrium diagram showed that rs2234130, rs710100, rs146514706, and rs1132339 were tagSNPs of the TNFAIP2 gene, and the alleles of rs2234130 included rs8126, rs3759571, rs3759573, rs2234130, rs749206, rs4369588, rs2234143, rs8176365, rs2234131, rs2403128, rs944000, rs1887940, rs2234133, rs4283165, and rs11160713. [file Image_1.jpeg]

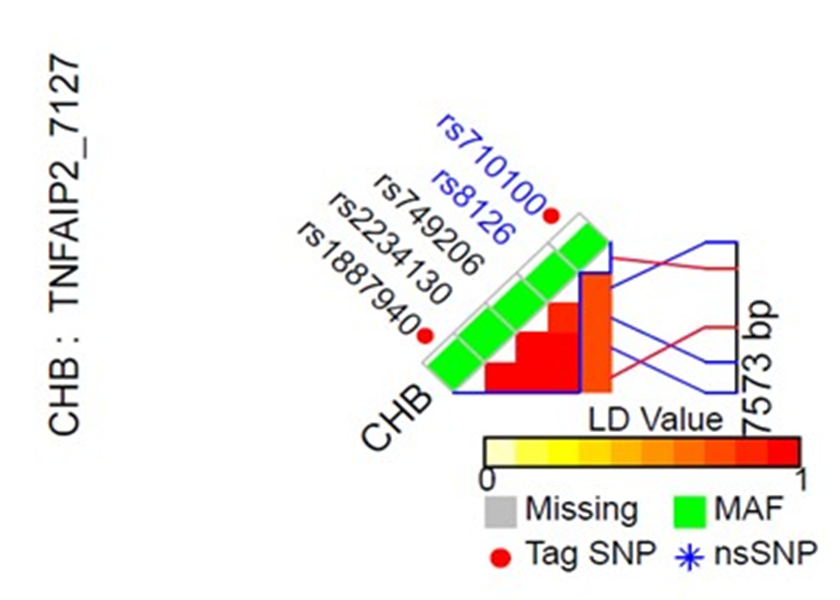

Supplement: Supplementary Figure 2 — Prediction diagram on TNFAIP2 tagSNPs by the NIH Snpinfo website. The functional tagSNPs of the TNFAIP2 gene were predicted by the NIH Snpinfo website. The parameters were set as Chinese Han population; minimum allele frequency >5%; frequency distribution r2 > 0.8. This prediction diagram showed that rs1887940 and rs710100 were tagSNPs of the TNFAIP2 gene, and the alleles of rs1887940 included rs8126, rs1887940, rs2234130, and rs749206. [file Image_2.jpeg]
